# Retinal transcriptome profiling at transcription start sites: a cap analysis of gene expression early after axonal injury

**DOI:** 10.1186/1471-2164-15-982

**Published:** 2014-11-18

**Authors:** Masayuki Yasuda, Yuji Tanaka, Koji M Nishiguchi, Morin Ryu, Satoru Tsuda, Kazuichi Maruyama, Toru Nakazawa

**Affiliations:** Department of Ophthalmology, Tohoku University Graduate School of Medicine, 1-1 Seiryo-machi, Aoba-ku, Sendai, Miyagi, 980-8574 Japan; Department of Retinal Disease Control, Tohoku University Graduate School of Medicine, 1-1 Seiryo-machi, Aoba-ku, Sendai, Miyagi, 980-8574 Japan; Department of Advanced Ophthalmic Medicine, Tohoku University Graduate School of Medicine, 1-1 Seiryo-machi, Aoba-ku, Sendai, Miyagi, 980-8574 Japan

**Keywords:** CAGE, Cap analysis of gene expression, Transcription start sites, Transcriptome, Axonal injury, Optic nerve crush, Retinal ganglion cells, RGC

## Abstract

**Background:**

Glaucoma is characterized by progressive loss of the visual field and death of retinal ganglion cells (RGCs), a process that is mediated, in part, by axonal injury. However, the molecular pathomechanisms linking RGC death and axonal injury remain largely unknown. Here, we examined these mechanisms with a cap analysis of gene expression (CAGE), which allows the comprehensive quantification of transcription initiation across the entire genome. We aimed to identify changes in gene expression patterns and to predict the resulting alterations in the protein network in the early phases of axonal injury in mice.

**Results:**

We performed optic nerve crush (ONC) in mice to model axonal injury. Two days after ONC, the retinas were isolated, RNA was extracted, and a CAGE library was constructed and sequenced. CAGE data for ONC eyes and sham-treated eyes was compared, revealing 180 differentially expressed genes. Among them, the *Bcat1* gene, involved in the catabolism of branched-chain amino acid transaminase, showed the largest change in expression (log2 fold-change = 6.70). In some differentially expressed genes, alternative transcription start sites were observed in the ONC eyes, highlighting the dynamism of transcription initiation in a state of disease. *In silico* pathway analysis predicted that ATF4 was the most significant upstream regulator orchestrating pathological processes after ONC. Its downstream candidate targets included *Ddit3,* which is known to induce cell death under endoplasmic reticulum stress. In addition, a regulatory network comprising IFNG, P38 MAPK, and TP53 was predicted to be involved in the induction of cell death.

**Conclusion:**

Through CAGE, we have identified differentially expressed genes that may account for the link between axonal injury and RGC death. Furthermore, an *in silico* pathway analysis provided a global view of alterations in the networks of key regulators of biological pathways that presumably take place in ONC. We thus believe that our study serves as a valuable resource to understand the molecular processes that define axonal injury-driven RGC death.

**Electronic supplementary material:**

The online version of this article (doi:10.1186/1471-2164-15-982) contains supplementary material, which is available to authorized users.

## Background

Glaucoma, one of the leading causes of blindness worldwide [1], is accompanied by unique progressive morphological changes in the optic nerve head, termed “glaucomatous optic neuropathy”. These changes are associated with characteristic patterns of visual field defects [2]. The contribution of elevated intraocular pressure (IOP) to glaucoma development and progression is well established, and currently available treatments have focused almost entirely on lowering IOP [3]. However, reported data have clearly demonstrated that even a substantial reduction in IOP cannot halt disease progression in many cases, which has led to increased attention to IOP-independent risk factors for glaucoma [4]. The understanding that glaucoma is a multifactorial disease has been solidified by strong clinical evidence suggesting that high myopia [5], age [6], reduced ocular blood flow [6], and axonal injury [7] may exacerbate glaucoma independently of IOP. Thus, in order to improve the management of glaucoma and mitigate the associated risk of blindness, it is important to improve our understanding of the pathologies that lead to deterioration in vision independently of IOP.

Axonal injury, possibly related to structural changes in the lamina cribrosa, has been proposed as an IOP-independent factor contributing to glaucoma [8–12]. Histopathological analysis has shown that mechanical stress on the axon bundles at the optic nerve head may occur in patients with glaucoma [13]. While such structural changes may be the consequence of age-related degenerative processes [14–17], factors such as ischemia [18, 19], inflammation [20, 21], and oxidative stress [22, 23] may also contribute. Moreover, axonal injury has been suggested to precede visual field defects in glaucoma patients [24]. However, little is known about the molecular events that link the injury to the axonal bundles and the death of RGCs (the proximal cause of glaucoma).

One of the most effective approaches to understand the molecular events that cause RGC death after axonal injury is comprehensive gene expression analysis using animal models. Most past studies that analyzed molecular events after axonal injury to the optic nerve in animal models took a targeted approach, in which one or, at most, a few molecules were selected for characterization [25]. Conversely, results obtained through microarray analysis have provided a list of many candidate genes that may be involved in the death of RGCs, providing a global view of change in gene transcription [26, 27]. However, microarray analyses rely on the hybridization of a set of known transcripts and are not as comprehensive as sequencing-based techniques [28]. In order to overcome this problem, we recently performed RNA sequencing (RNA-seq) using a next-generation sequencer on the eyes of mice which had undergone optic nerve crush (ONC) [29]. Profiling gene expression in these eyes uncovered a number of differentially expressed genes (DEGs) that may characterize ongoing biological processes in the ONC. Nevertheless, this technique relies on the comprehensive sequencing of random fragments of RNA with little attention to transcription start sites (TSSs) [28]. From this perspective, cap analysis of gene expression (CAGE) can be considered a complementary technique to RNA-seq, as CAGE analysis depends on the construction of full-length cDNA libraries and counting of the short tags at the 5′ end of the transcripts [30, 31]. In this way, the distribution of TSSs and networks of gene transcription can be studied comprehensively and quantitatively on a genome-wide scale. CAGE analysis is more efficient than conventional Rapid Amplification of cDNA End or EST analysis, as high throughput is possible at a relatively low cost [32]. The usefulness and power of this technique have been widely recognized through its contribution to the “Encyclopedia of DNA Elements” (ENCODE) project, which elucidated the global distribution of promoter areas in the human genome and the regulatory network of transcription factors [33, 34].

In this study, we applied CAGE analysis to retinal samples 2 days after ONC, in order to comprehensively study changes in gene transcription at TSSs. Using CAGE data, we attempted to determine dynamic changes in the regulation of the transcriptional network mediating RGC death after axonal injury.

## Methods

### Animals

Forty-six C57BL/6 mice (male, 12 weeks old; SLC, Hamamatsu, Japan) were used in this study. The surgical procedures were performed under deep anesthesia, which used intramuscular administration of a mixture of ketamine (100 mg/kg) and xylazine (9 mg/kg). All animals were maintained and handled in accordance with the guidelines of the ARVO Statement for the Use of Animals in Ophthalmic and Vision Research and the guidelines from the Declaration of Helsinki. All experimental procedures described in the present study were approved by the Ethics Committee for Animal Experiments at Tohoku University Graduate School of Medicine.

### Induction of axonal injury

Axonal injury was induced by ONC as previously described [25, 35]. Briefly, the optic nerve was exposed, and then crushed 2 mm posterior to the globe with fine forceps for 10 seconds. We confirmed that retinal blood circulation was normal with a fundus examination, and then applied antibiotic ointment. We also performed sham operations on a separate group of mice, in which the procedure was similar but the optic nerve was not crushed.

### RNA preparation

Total RNA was purified from each retinal sample as previously described [29]. Two days after surgery, the retinas were extracted and immediately immersed in RNAlater RNA Stabilization Reagent (Qiagen, Valencia, CA). The retinas were then homogenized in Qiazol (Qiagen) with a pestle homogenizer, and total RNA was extracted from the homogenized mixture with a miRNeasy mini kit (Qiagen). The resulting 46 individual samples (23 in each group) were then assessed with a spectrophotometer to estimate their total RNA concentration (NanoDrop 2000c, Thermo Scientific). To prepare the RNA samples for CAGE, fixed quantities of RNA were taken from six samples and combined into a single sample, in order to minimize the influence of individual variations in the mice [29]. The quality of these six combined RNA samples was then assessed with an Agilent 2100 Bioanalyzer (Agilent Technologies). The RNA integrity number of each combined sample used for the cDNA preparation is shown in Additional file 1.

### CAGE library preparation and sequencing

A CAGE cDNA library was prepared as previously described [36, 37], with minor adaptations for the Illumina sequencer platform [38]. Five μg of RNA from each retinal sample were used to synthesize single-strand cDNA. The cDNA was then reverse-transcribed with a random primer N6 primer (5′-TCTNNNNNN-3′). The resulting cDNA/RNA hybrids were oxidized with NaIO_4_ in order to open the diol at the 5′ end on the cap structure, and the diol group at the 3′ end of each RNA strand. The derived oxidized dialdehyde of the cap site and 3′ ends of the RNA strands were biotinylated with biotin (long arm) hydrazide (Vector Laboratories) and treated with RNaseONE (Promega) in order to remove the 3′ end of each RNA strand and the biotinylated cap when cDNA failed to reach the 5′ ends. The biotinylated 5′ end of each RNA strand was selectively trapped with magnetic streptavidin beads (Dynabeads MyOne Streptavidin C1 beads, Life Technologies). The captured cDNA was then released from the beads with RNaseONE treatment, and the single-strand cDNA was purified with Agencourt AMPure XP (Beckman Coulter) according to the manufacturer’s instructions. A 5′ linker with a barcoded sequence was ligated to the 5′ end of the cDNA. The cDNA was purified with Agencourt AMPure XP, and then a 3′ linker containing an Illumina adapter sequence was ligated to it. The cDNA was again purified with Agencourt AMPure XP, followed by treatment with Shrimp Alkaline Phosphatase (Affymetrics) and USER (NEB) to restrict the upper strand of the 3′ linker. Second-strand cDNA was synthesized with a second primer consisting of another Illumina adapter sequence. After Exonuclease I (NEB) treatment, the resulting second-strand cDNA was purified with Agencourt AMPure XP. The cDNA concentration of the final product was determined with a Quant-iT PicoGreen dsDNA Assay Kit (Life Technologies).

Cluster generation of the cDNA was performed with a cBot fluidics device and the Illumina cBot software. One lane of the flow cell was used for sequencing with four-color DNA Sequencing-By-Synthesis (SBS) technology using the Illumina HiSeq 2000 (Illumina, San Diego, CA). The sequencing run and the base call analysis were performed according to the manufacturer's protocol with a TruSeq SBS kit v3-HS (Illumina). After the sequencing, raw sequence data were generated by Illumina RTA 1.12.4.2 and CASAVA-1.8.2. The sequence data were recorded as FASTQ files. All CAGE sequence data are available under the accession number DRA002410.

### CAGE data processing and differential expression analysis

For processing and analysis of sequenced CAGE data, we used an integrated platform provided by the Data Analysis Center of the Cell Innovation Program (http://cell-innovation.nig.ac.jp). The overall workflow is shown in Additional file 2. Primary data processing of the sequenced data was performed with the nAnT-iCAGE pipeline [36]. Read alignment and sequence mapping were performed with BWA software [39]. All sequence reads were mapped to the reference genome (NCBI37/mm9). The mapped data were recorded in the SAM format file, and converted to BAM files with SAMtools [40]. The mapping quality was assessed with SAMStat software [41]. All the processed data in the BAM files were imported to a RECLU pipeline [42]. The RECLU is a method of implementing clustering, differential expression analyses, and motif discovery analyses. The core steps of the RECLU pipeline include clustering of individual TSSs with a modified version of the Paraclu algorithm, merging overlapping peaks in different replicates and applying an irreproducible discovery analysis (IDR) to select reproducible peaks [42–44]. In order to apply the Paraclu methods to CAGE datasets, the mapped reads were converted into the CAGE-defined transcriptional start sites format. The mapped reads at each site were counted with SAMtools. In the modified Paraclu program, a normalized tag per million (TPM) per base threshold was used and clusters with < 0.1 TPM per base were omitted [42]. Both the original and modified Paraclu programs define clusters as maximal scoring segments, found by varying a density parameter (d) [43, 45]. In this study, the stability of each cluster, defined as max d/min d, was calculated with the modified Paraclu program. If a particular segment had maximal scoring over a large range of values for d, we considered it to be a stable cluster [42]. The IDR is a reproducibility criterion analogous to the false discovery rate (FDR) [44]. We only selected clusters with an IDR < 0.1. Differential gene expression analysis was performed with the Bioconductor package ‘edgeR’ [46]. DEGs were defined as having an FDR adjusted *P*-value < 0.05 along with absolute fold-change > 1.5. The AMD [47], GLAM2 [48], Weeder [49], and DREME [50] programs were used to identify motifs [42], and the Tomtom program [51] was used to compare standard motif representations in the JASPAR core database [52].

### Quantitative real-time PCR

Ten samples of purified RNA (n = 5 in each group) were used in a quantitative real-time PCR (qRT-PCR) analysis. Total RNA (200 ng per sample) from each sample was reverse-transcribed into cDNA using SuperScript III (Invitrogen Life Technologies, Carlsbad, CA). QRT-PCR was then performed with a 7500 Fast Real-Time PCR System (Applied Biosystems, Foster City, CA) as previously described [53]. For each 20 μl reaction the following were used: 10 μl TaqMan Fast Universal PCR Master Mix (Applied Biosystems, Foster City, CA), 1 μl Taqman probe, 1 μl template DNA, and 8 μl DEPC water. For a relative comparison of gene expression, we analyzed the results of the qRT-PCR data with the comparative Ct method (2^- ∆∆CT^), normalized to *Gapdh*, an endogenous control. All Taqman probes used for these reactions are listed in Additional file 3.

### Pathway analysis

*In silico* pathway analyses were performed with Ingenuity Pathway Analysis (IPA, Ingenuity Systems, Redwood City, CA) as previously described [29, 54, 55]. The DEG datasets were uploaded to the IPA application and mapped to the Ingenuity Pathways Knowledge Base (IPKB). Each gene identifier was then mapped to its corresponding IPKB. Duplication of genes in the DEG datasets was resolved by selecting the gene with the maximum fold-change value [56]. Networks of these genes were generated based on their connectivity. The significance of the association between the datasets and biofunctions was measured as the ratio of the number of genes from the dataset that mapped to the pathway divided by the total number of genes in that pathway. An upstream regulator analysis was performed to compare DEGs in the datasets to those known to be regulated by a given upstream regulator. Based on the concordance between them, an activation score was assigned, showing whether a potential transcriptional regulator was in an “activated” (z-score ≥ 2), “inhibited” (z-score ≤ -2), or uncertain state. The regulator effects analysis was also performed with IPA, in order to discover relationships between upstream regulators and downstream functions and diseases. Only regulators and downstream functions and diseases with *P* < 0.05 and |z- score| ≥ 2 were used in this analysis. The regulator effects algorithm connects upstream regulators, dataset molecules and downstream functions or diseases affected in the dataset to generate a hypothesis that can explain how the upstream regulators affect the downstream target molecule expression and the impact of the molecular expression on functions and diseases. The consistency score, a measure of the causal consistency and density of connection in a regulator effects network, was also calculated with IPA.

### Statistical analysis

Differential gene expression analysis of CAGE data was performed with the R Bioconductor package ‘edgeR’ integrated in the RECLU pipeline, as described above. *P*-values were adjusted for multiplicity with Benjamini-Hochberg correction with edgeR. Genes with adjusted *P*-values < 0.05 and absolute fold-change > 1.5 were considered DEGs in the CAGE analysis.

QRT-PCR data were analyzed with the Welch’s *t*-test. Statistical analysis of the qRT-PCR data was performed with R software (version 3.1.0) [57]. The significance of the pathway analysis was calculated with Fisher’s exact test in the IPA application. If the *P*-values for qRT-PCR and IPA were less than 0.05, the results were considered statistically significant.

## Results

### Validation of the CAGE data

Previously, we obtained experimental data indicating that RGC death starts approximately 3 days after ONC in mice [25]. As our primary interest was to use the CAGE data to search for therapeutic targets for RGC preservation, we examined changes in the retina on 2 days after ONC (Day 2), in order to observe the molecular events preceding the actual death of the cells. Total RNA was extracted from 3 independent retinal samples, each of which was derived from 6 retinas extracted from 6 different mice. CAGE was then performed on these 3 samples. The analysis workflow for the data derived from CAGE is well established [42]. An outline of the process is shown in Additional file 2.

**Figure 1:**
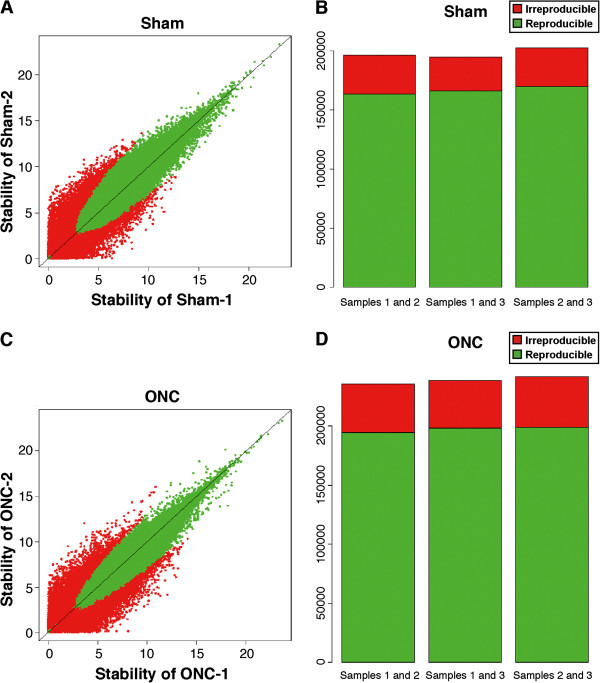
**Stability and reproducibility of the biological replicates used for CAGE.** The scatter plots show the correlation between expression values in replicates 1 and 2 in both the sham **(A)** and ONC **(C)** groups. Stability is a score used for clustering reproducible TSSs in multiple replicates. Green dots indicate high reproducibility and red dots indicate low reproducibility. The bar graphs represent a reproducibility evaluation for each pair of replicates in the sham **(B)** and ONC **(D)** groups. Green indicates TSS clusters with high reproducibility between the replicates. Red indicates TSS clusters with low reproducibility between the replicates.

Before beginning the analysis of the CAGE data, we evaluated its integrity (Additional file 1). We found that, in all samples, at least 89.8% of the sequence data mapped to the reference genome (NCBI37/mm9), assuring that the overall quality of the CAGE data was high. Furthermore, the MAPQ value, which represents the mapping quality of each sample, was more than 30 in at least 75.3% of the mapped data. Finally, we tested the gross consistency of the data among biological samples by comparing pairs of expression data derived from each treatment group using the modified Paraclu and the IDR program in the RECLU pipeline. The IDR analysis was used to quantitatively measure consistency between biological replicates and to select reproducible signals. These analyses revealed that the expression patterns in sets of data derived from independent samples taken from the same group were highly similar (Figure 1), assuring the high integrity of the CAGE datasets. Taken together, the quality of the data thus met the reliability requirements for downstream processing [41, 58].

### Comparative analysis of DEGs

**Figure 2:**
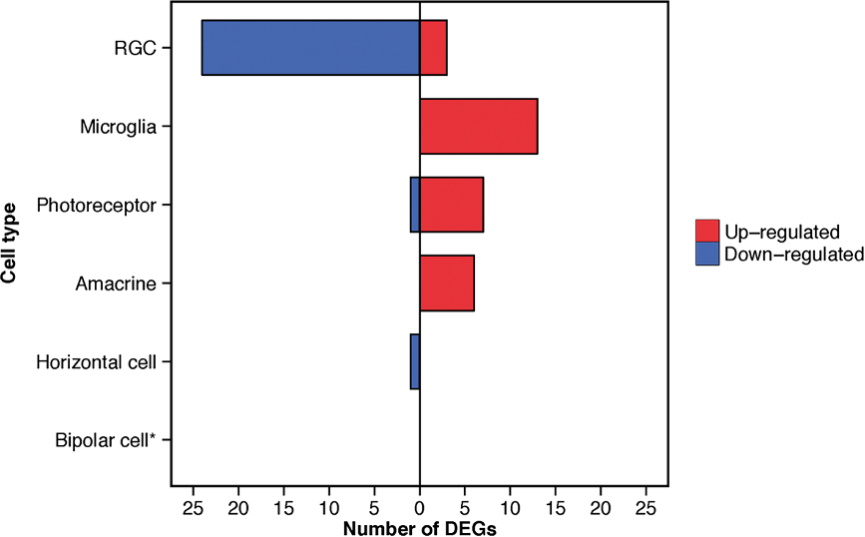
**Overall reduction in the RGC-specific transcriptome.** The horizontal histogram shows the number of DEGs in each retinal cell type [59]. Red: up-regulated, Blue: down-regulated. *No DEGs specific to bipolar cells were detected in this study.

After assessing the quality of the CAGE data and defining the TSSs of our data, we used the edgeR package to perform a differential expression analysis of the transcripts at each TSS in the eyes subjected to ONC and the eyes that received a sham procedure. Consequently, we identified 400 differentially expressed TSSs, which included 180 annotated (Additional file 4) and 220 unannotated TSS clusters (Additional file 5). We further divided the 180 annotated DEGs into 6 groups of genes based on their retinal cell type, i.e., RGCs, microglia, photoreceptors, amacrine cells, horizontal cells, and bipolar cells, using published microarray gene expression data [59]. According to the microarray database, 55 of 180 DEGs were expressed more specifically in one of the first 5 of these retinal cell types (Additional file 6). However, no DEGs specific to bipolar cells were detected in this study. We found that 88.9% of the genes that were relatively specific to RGCs were down-regulated in the ONC retinas, which was in sharp contrast with other cell types studied (Figure 2). This pattern of expression is, in fact, in good agreement with the expected reduction of transcription in severely injured RGCs [60, 61], further validating the biological accuracy of the CAGE data.

**Figure 3:**
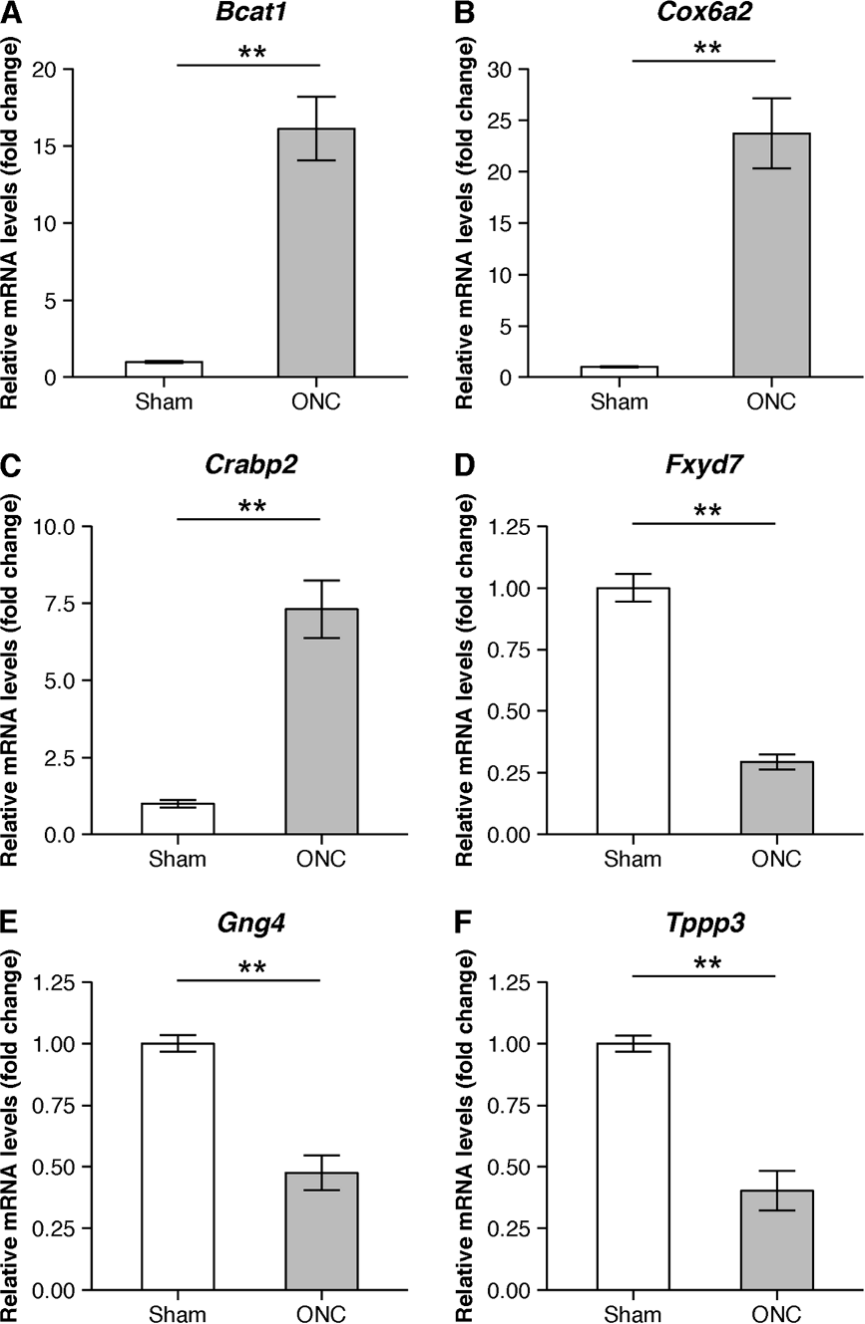
**Validation of selected DEGs with qRT-PCR.** Transcriptional changes in six selected DEGs were validated with qRT-PCR **(A-F)**. The graphs show the level of mRNA expression in the ONC group relative to the sham group. The average expression for sham group was normalized as a 1.0-fold change. Values are mean ± SD (n = 5 in each group, **P < 0.01).

Next, we determined if there were specific correlations between the level of transcripts at a given TSS and the level of transcripts detectable by qRT-PCR. The expression of six randomly selected highly significant DEGs (*Bcat1*, *Cox6a2*, *Crabp2*, *Fxyd7*, *Gng4* and *Tppp3*) was quantified by choosing the primer pair downstream of the TSS of interest. Conventional qRT-PCR showed a significant difference in expression in the six genes in the sham-treated and ONC samples (Figure 3). This was consistent with the differential expression patterns observed in the CAGE data.

The type of TSS could also be further classified based on the distribution of the mapped tags forming two different types of clusters with sharp TATA-box-associated promoters (top peaks) and broad CpG-associated promoters (bottom peaks) ([62]) (Additional file 7). The 10 most significantly up- or down-regulated DEGs at the top and bottom peaks are shown in Table 1. We found that 45% of the top 10 up-regulated DEGs and 55% of the top 10 down-regulated DEGs at either or both peaks after ONC were also up-regulated in the RNAseq data derived from the same model [29]. Meanwhile, 75% of the up-regulated DEGs and 45% of the down-regulated DEGs were also identified as DEGs in the microarray data derived from mouse retinas 3 days after ONC, one day later than the current study [27].

**Table 1:** **Top 10 up- and down-regulated genes in each peak type after ONC**

Gene	Gene accession	Log _2_ fold-change	Adjusted *P*-value
**Up-regulated at top peaks**			
*Bcat1*	NM_007532	6.70	7.80E-04
*Sprr1a*	NM_009264	4.66	1.80E-23
*Mmp12*	NM_008605	4.36	1.70E-39
*Adcyap1*	NM_009625	4.27	7.10E-08
*Zfp275*	NM_001160229	3.88	3.50E-02
*Ecel1*	NM_001277925	3.60	2.00E-07
*Crabp2*	NM_007759	3.56	2.60E-10
*Arhgef2*	NM_001198911	3.53	2.40E-02
*Gal*	NM_010253	3.49	7.60E-07
*Asns*	NM_012055	3.44	3.50E-06
**Up-regulated at bottom peaks**			
*Sprr1a*	NM_009264	4.76	3.90E-31
*Mmp12*	NM_008605	4.14	1.80E-51
*Ecel1*	NM_001277925	3.74	1.60E-20
*Crabp2*	NM_007759	3.55	2.40E-11
*Gal*	NM_010253	3.49	1.80E-18
*Cox6a2*	NM_009943	3.25	2.50E-11
*Thoc7*	NM_001013578	3.21	9.10E-03
*Zfand2b*	NM_026846	3.20	2.00E-04
*Tnfrsf12a*	NM_001161746	2.95	6.60E-06
*Arhgef2*	NM_001198911	2.92	3.00E-09
**Down-regulated at top peaks**			
*Gng4*	NM_010317	-6.39	7.50E-03
*Ak4*	NM_009647	-3.45	2.50E-02
*Ndufa13*	NM_023312	-3.02	3.10E-02
*Tusc5*	NM_177709	-2.40	1.70E-04
*Nrgn*	NM_022029	-2.20	8.00E-03
*Fxyd7*	NM_022007	-2.15	7.50E-12
*Ctxn3*	NM_001134697	-2.09	4.60E-03
*Tppp3*	NM_026481	-1.58	3.00E-02
*Pvalb*	NM_013645	-1.53	1.80E-05
*Sncg*	NM_011430	-1.46	4.60E-03
**Down-regulated at bottom peaks**			
*Scarna9*	NR_028568	-2.66	3.10E-02
*Tusc5*	NM_177709	-2.43	9.10E-06
*Cnn3*	NM_028044	-2.41	8.60E-03
*Fxyd7*	NM_022007	-1.62	4.50E-13
*Ctxn3*	NM_001134697	-1.58	1.80E-04
*Rasgrp2*	NM_011242	-1.47	2.20E-07
*Tmsb10*	NM_001039392	-1.44	4.50E-05
*Bcl2*	NM_009741	-1.43	6.80E-03
*1500009C09Rik*	NR_037698	-1.41	4.30E-05
*Oasl1*	NM_145209	-1.34	4.30E-05

Differences were considered significant with an adjusted *P*-value < 0.05 and |fold-change| > 1.5.

In some DEGs, we found evidence of the emergence of alternative promoters after ONC. For example, in the sham-treated eyes, transcription of *Tnfrsf12a* was almost exclusively dependent on the TSS of exon 1 of the reference transcript (NM_013749). However, in the retinal samples from the ONC eyes, a cryptic promoter embedded around exon 2 emerged as an equally dominant TSS (Additional file 8). We also performed a promoter database search with ZENBU [63], which contains FANTOM5 (Functional Annotation of Mammalian Genome 5) datasets [64]. According to ZENBU, the use of this *Tnfrsf12a* promoter has been already recognized in hepatocyte and Schwann cells.

Furthermore, we discovered that 34 of the 220 unannotated TSS clusters differentially expressed 2 days after ONC did not have any CAGE peaks in ZENBU (Additional file 5). These 34 TSS clusters may therefore include novel promoters and/or novel long non-coding RNAs specifically affected by axonal injury. We investigated the tissue specificity of the remaining 186 unannotated TSSs registered in ZENBU. However the database did not contain specific expression profile data for the retina, but only for the whole eye. According to ZENBU, 6 of the 186 unannotated TSSs were specifically expressed in the murine eye (Additional file 5).

### *In silico*pathway analysis of the DEGs

An *in silico* pathway analysis of the bioinformatics of the 180 DEGs revealed five potential biological processes that occurred differentially in the retinas of the ONC and sham-treated groups. Among these processes, 42 DEGs contributing to the “Cell Death and Survival” pathway emerged as the most significant (Table 2). Since one of our main goals was to understand the molecular network that mediates the death of RGCs in axonal injury, we took a deeper look into the DEGs that contributed to this pathway (Additional file 9). Based on the information in Additional files 4 and 9, we generated a file that lists the DEGs in the “Cell Death and Survival” pathways, and indicates their involvement in the “Cell death” and “Cell survival” pathways, as well as their gene expression changes 2 days after ONC (Additional file 10). We found that the endoplasmic reticulum (ER) stress-related genes *Atf3*, *Ddit3*, *Egr1* and *Jun* [65, 66], which belong to the cell death pathway, were up-regulated 2 days after ONC (Additional file 10). *Bbc3*, a pro-apoptotic BH3-only gene [67] was also up-regulated (Additional file 4). Additionally, *Bcl2* [68], *Park7* [69] and *Serpinf1* [70], which belong to the cell survival pathway, had altered expression 2 days after ONC: *Bcl2* was down-regulated, whereas *Park7* and *Serpinf1* were up-regulated (Additional file 10).

**Table 2:** **Top 5 molecular and cellular biological pathways significantly altered after ONC**

Category	*P*-value	Number of molecules
Cell death and survival	1.99E-09 - 7.11E-03	42
Cellular development	1.51E-05 - 7.11E-03	24
Cellular growth and proliferation	1.51E-05 - 7.11E-03	32
Cell-to-cell signaling and interaction	2.11E-05 - 7.11E-03	30
Cellular function and maintenance	2.18E-05 - 7.11E-03	32

Significances were calculated with Fisher’s exact test.

Differences were considered significant at the *P* < 0.05 level.

**Figure 4:**
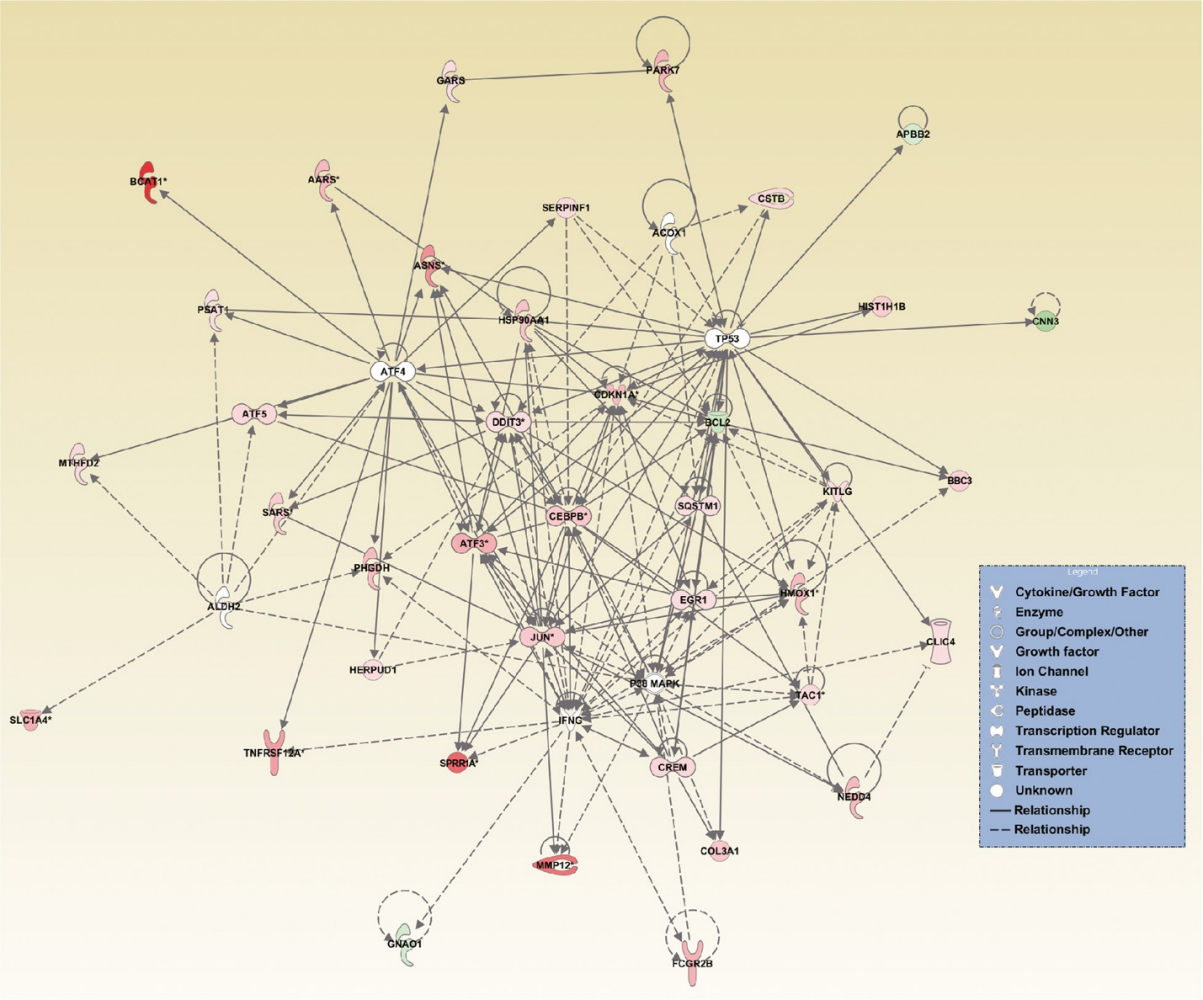
**Predicted protein interaction networks involved in axonal injury.** Prediction of upstream regulators was performed using IPA. The predicted upstream regulators (white nodes) were ATF4, IFNG, TP53, P38MAPK, ALDH2, and ACOX1. The targets of these upstream regulators are also displayed to illustrate the alteration in the interaction networks after ONC. Solid lines represent a direct interaction between two genes, whereas dotted lines represent an indirect relationship. The length of a line reflects the strength of reported evidence supporting the node-to-node relationship. The shapes of the nodes represent the different known biological roles of each of these molecules, as shown in the lower right inset. Red indicates up-regulated genes, green indicates down-regulated genes, and white indicates genes that were not annotated in these CAGE results, but that formed part of the network. *:targets that were duplicated in the dataset.

For the purpose of selecting therapeutic targets, it is useful to understand the hierarchy of a defined molecular network. Therefore, we performed an additional *in silico* analysis to identify the key upstream regulators that govern these networks [71]. This analysis revealed six important upstream regulators, each predicted to act upon different sets of target molecules, likely mediating different biological effects (Figure 4, Table 3). Additionally, the regulator effects analysis predicted that a regulatory network comprising IFNG, P38 MAPK, and TP53 was involved in cellular death (Additional file 11).

**Table 3:** **Predicted upstream regulators after ONC**

Name	Predicted change	Activation z-score	*P*-value of overlap	Target molecules in dataset
ATF4	Activated	3.14	6.10E-18	AARS, ASNS, ATF3, ATF5, BCAT1, CDKN1A, CEBPB, DDIT3, GARS, HERPUD1, MTHFD2, PSAT1, SARS, SERPINF1, TNFRSF12A
IFNG	Activated	2.64	2.05E-04	BCL2, CDKN1A, CEBPB, CLIC4, CREM, DDIT3, FCGR2B, GNAO1, MMP12, SPRR1A, TAC1
P38 MAPK	Activated	2.22	2.42E-06	BBC3, CDKN1A, EGR1, HMOX1, JUN, NEDD4
TP53	Activated	2.14	6.99E-05	APBB2, ATF3, BBC3, BCL2, CDKN1A, CLIC4, CNN3, COL3A1, CSTB, HIST1H1B, HMOX1, HSP90AA1, KITLG, PARK7
ALDH2	Inhibited	-2.22	1.94E-07	ATF5, MTHFD2, PHGDH, PSAT1, SLC1A4
ACOX1	Inhibited	-2.00	1.33E-02	CDKN1A, CSTB, DDIT3, SQSTM1

Data were analyzed with Fisher’s exact test. Differences were considered significant with a *P*-value < 0.05 and |z-score| ≥ 2. The activation z-score was used to infer the likely activation states of upstream regulators based on a comparison with a model that assigns random regulation directions. The *P*-value overlap, which indicates likely upstream regulators, represents the significance of the overlap between the dataset genes identified here and known targets of transcriptional regulators.

An alternative way to search for key upstream transcription factors defining the pathology of ONC eyes is to explore the dominant binding motifs embedded near the TSS. For this purpose, we took advantage of a previously established program [51] to predict the motifs to which the transcription factor bound. Target motifs were recovered for each of the four patterns of quantitative change in transcription (Table 4 and Additional file 12). This information predicted that 5 transcription factors (SP1, PAX4, RREB1, Tal1/Gata1 complex and NFATC2) interacted with the recovered motifs and exerted biological effects contributory to the process of axonal injury.

**Table 4:** **List of predicted motif sequences associated with axonal injury**

Motif no.	Consensus	Foreground	Background	*P*-value	Known motifs (*P*-value)
**Up-regulated at top peaks**					
AMD_001	YNRNAGGTGT	21	101	2.50E-06	NA
AMD_002	CCTNDGNNNGAG	24	167	7.79E-05	NA
AMD_003	WGAGNNTTACCNS	20	91	2.65E-06	NA
AMD_005	GNNNGTGNTGATGNC	19	92	1.30E-05	NA
AMD_006	YDNWNATTCHTAGGYNA	16	102	1.61E-03	NA
AMD_007	CNNNMAGARTNNTTGNMNW	22	115	3.75E-06	NA
AMD_009	ACGNNATAYWNNNA	16	92	5.85E-04	NA
GLAM2_001	SCBCCCBCCCCYCCCCCNCCCB	34	330	2.24E-05	SP1 (5.25E-08), Pax4 (6.97E-07), RREB1 (5.84E-06)
**Up-regulated at bottom peaks**					
AMD_003	CTGSNYNNAGA	22	128	4.80E-06	NA
AMD_004	SNYAGGWGTCATK	18	87	9.50E-06	NA
AMD_006	ATCNNNNNNBCCAM	20	106	6.28E-06	Tal1::Gata1 (3.02E-06)
AMD_009	CTGNNNNNNNNNTNNANAKANNNA	21	162	5.58E-04	NA
GLAM2_004	CCBCCYCCTCCHBHCHCCC	33	371	1.07E-04	Pax4 (1.56E-05), SP1 (2.93E-05)
**Down-regulated at top peaks**					
AMD_002	WTCAATGAKWTACANTGWWMW	20	193	5.23E-03	NA
AMD_003	SANKWAMAMTGARAAAMAYM	23	237	3.82E-03	NA
GLAM2_005	TTCTTTYTTBTTYBTYTYYHTTTYT	30	360	8.54E-04	NA
**Down-regulated at bottom peaks**					
GLAM2_006	AAAMATGRAAAATRANAAAAANCAMA	24	327	0	NFATC2 (6.39E-05)
DREME_001	ACTCATCTA	13	56	2.14E-06	NA
DREME_002	AAAACCACACTGTA	18	124	1.42E-06	NA
DREME_003	ATGAGTTAC	12	38	5.11E-07	NA
DREME_004	AGTTACACTGAA	14	83	1.65E-05	NA
DREME_006	TACACTGTTCTACA	12	87	5.72E-04	NA
DREME_007	ATTCGTTGG	8	22	3.70E-05	NA
DREME_008	ATATTTCA	16	99	3.37E-06	NA
DREME_009	AATGAGAAAC	14	74	5.10E-06	NA
DREME_010	TCACTAAAA	14	104	1.62E-04	NA
DREME_011	ACTGTAGGA	15	82	2.43E-06	NA
DREME_012	AAACGGGATT	10	73	2.26E-03	NA
DREME_013	HTATGAA	15	78	1.40E-06	NA
DREME_014	ATATGTTC	16	102	4.82E-06	NA

Foreground values indicate the number of occurrences of a motif sequence in DEGs after ONC.

Background values indicate the number of occurrences of a motif sequence in non-DEGs after ONC.

## Discussion

In this study, CAGE analysis was performed on retinal RNA samples collected 2 days after the ONC procedure, to comprehensively and quantitatively compare TSSs scattered throughout the genome and elucidate the molecular pathomechanisms underlying the RGC death induced by axonal injury. Through the use of a unique analysis pipeline, our study identified a list of DEGs with high value.

On a global level, we observed that transcription of DEGs in the RGCs was generally depressed. This pattern of expression change was unique to DEGs in the RGCs, as DEGs in the other cell types (microglia, photoreceptors, and amacrine cells) showed the opposite trend. In fact, we were rather surprised to see up-regulation of genes specific to photoreceptors and amacrine cells. We suspect that inflammatory soluble factor production may have exerted an off-target stimulatory effect, as a paradoxical increase in retinal function has been reported in the early phases of intraocular inflammation [72]. Meanwhile, it was not surprising to observe the up-regulation of all 14 DEGs specifically expressed in microglia, as these cells have an important role in scavenging dying neurons [73]. One of these 14 DEGs, *Clic1,* is involved in the production of reactive oxygen species [74], which could be a key mediator of RGC death, as oxidative stress is known to contribute to the pathology of axonal injury [75]. In this study, we only examined changes in gene expression 2 days after ONC because, unlike the sham group, the number of RGCs significantly decreases at later time points in ONC group, which can complicate the direct comparison of gene expression between the two groups. However, it has been reported that inflammation and oxidative stress, as well as ER stress, are more activated at later time points after axonal injury [75, 76]. Therefore, it is also important to evaluate changes in transcriptome profiles in these later stages, and we hope to investigate them in a future study using a different approach.

Among the 180 DEGs, *Bcat1* showed the largest expression difference (Table 1). The validity of this CAGE data was verified with conventional qRT-PCR, which showed that expression increased in the ONC eyes by ~16.1-fold (Figure 3). This gene is therefore highly interesting as a therapeutic target, but its involvement in axonal injury and RGC death has not yet been reported. *Bcat1* encodes the enzyme branched-chain amino acid transaminase and is the target of c-Myc. It can reportedly induce cell death by causing the production of excessive branched-chain keto acids through transamination [77]. It is also possible that this gene mediates the death of the RGCs via a similar mechanism.

Our *in silico* pathway analysis revealed that the “Cell Death and Survival” pathway was the most significant biological process in the ONC retinas (Table 2). Among relevant DEGs, genes involved in ER stress eventually leading to cell death, such as *Atf3* and *Ddit3,* were up-regulated 2 days after ONC (Additional files 4 and 10). It has been reported that *Ddit3* up-regulates mRNA expression of *Bbc3*, a cell death-related gene [78], and also down-regulates mRNA expression of *Bcl2*, a cell survival-related gene [79]. Up-regulation of *Bbc3* and down-regulation of *Bcl2* were confirmed in this study (Additional file 4) and may be caused by the increased transcription of *Ddit*3 and activation of ER stress as a mechanism of RGC death after ONC.

We also found that *Serpinf1* (aka *Pedf*) was a cell survival-related DEG (Additional file 10). *Serpinf1* reportedly plays a neuroprotective role in ONC [80]. *Park7* was also classified as a cell survival-related DEG (Additional file 10). Previous reports showed that the translated protein of *Park7* increased 4 days after ONC in rats [81] and that mutations in *Park7* were associated with Parkinson’s disease (PD) [82]. Animal models of PD have suggested that the translated protein exerts an anti-oxidative effect that leads to neuroprotection [83]. While the role of *Park7* in the pathology of ONC is still unclear, it is possible that a similar anti-oxidative mechanism may protect the RGCs from axonal injury-induced death.

The significance of our findings was reinforced by the *in silico* identification of networks of DEGs and the prediction of the most significant upstream regulators for each network, including the prediction that ATF4 was an up-stream regulator of the ER stress pathway (Table 3). It is conceivable that ATF4 could increase the transcription of ER stress-related genes, including *Aft3* and *Ddit3*, which may ultimately promote RGC death [84]. We found similar up-regulation of ER stress-related genes in RNA-seq data obtained with a similar experimental design comparing ONC and sham-treated eyes [84]. Interestingly, *Bcat1* was also included as a target of ATF4. The list of predicted upstream regulators of altered pathways also included P38MAPK and TP53, both implicated in RGC death after ONC [85, 86].

In addition, we performed a motif discovery analysis and determined that the SP1 (Sp1 transcription factor) motif was differentially up-regulated at bottom peaks (Table 4). SP1 is a zinc-finger transcription factor that binds to GC-rich elements [87, 88]. It has been reported to regulate the transcription of damage-induced neuronal endopeptidase (DINE) through interaction with ATF3, c-Jun, and STAT3 [89]. DINE is also known as *Ecel1* [90], which was up-regulated after ONC in this study. Therefore, SP1 may be implicated in transcription regulation after axonal injury.

Despite CAGE and RNA-seq using clearly different quantitative methods to evaluate gene expression, we found that they had a certain degree of commonality in their final output when identifying DEGs. However, CAGE did provide us with a unique insight into differences in TSSs, especially through its analysis of the differential use of multiple promoters within a given gene, and through the discovery of the associated DNA-binding motifs. However, at this point, we do not know which comprehensive gene expression database is more useful for selecting therapeutic targets. It may be that DEGs commonly observed in both the CAGE- and RNA-seq-derived datasets will yield the best targets. Nevertheless, conclusions on this point cannot be drawn until the functional characterization of a number of candidate genes from both groups is completed.

## Conclusions

In summary, CAGE analysis followed by *in silico* molecular network analysis using retinal samples from a mouse ONC model revealed a list of DEGs partly matching a list previously identified with RNA-seq data [29]. A detailed analysis of TSSs provided us with a wealth of unique information on the differential use of promoters and the associated DNA binding motifs. As a next step, we aim to develop a high throughput pipeline to enable the efficient prioritization of candidate genes, using the current CAGE dataset to search for novel drug targets.

## Electronic supplementary material

Additional file 1: **Summary of mapping statistics from SAMStat output.** The pie charts show the number of sequence alignments in various mapping quality (MAPQ) intervals and the number of unmapped sequences. The percentage and number of alignments in each category is given in brackets. Red indicates reads with a high mapping accuracy (MAPQ > 30). Black indicates unmapped reads. The RNA integrity number (RIN) of each sample is shown after the sample name. (TIFF 1 MB)

Additional file 2: **CAGE data analysis workflow.** The workflow has two phases: primary data processing followed by clustering and analysis. During the first phase, the sequence data is evaluated for quality, filtered, mapped, and converted to an annotation file with a nAnT-iCAGE pipeline. During the second phase, the file is fed into the analytical pipeline, where the analysis of differential expression and motif discovery are carried out with the RECLU pipeline. (TIFF 163 KB)

Additional file 3: **List of Taqman probes used in this study.** (PDF 44 KB)

Additional file 4: **List of DEGs after ONC.** (XLSX 65 KB)

Additional file 5: **List of unannotated TSS clusters differentially expressed after ONC.** (XLSX 69 KB)

Additional file 6: **List of expression changes in the retinal cell-specific transcriptome.** (XLSX 50 KB)

Additional file 7: **Example of top and bottom peaks of TSS clusters.** Genome Explorer views of *Efemp1* and *Eif1* genes are shown here, representing TSS clusters with a top peak (A) and a bottom peak (B), defined with a modified Paraclu program. Based on the distribution of the mapped tags, TSS clusters show two types of structures, sharp with a high peak (termed “top”) and broad with a low peak (termed “bottom”) [42]. The red frame indicates a top peak. The blue frame indicates a bottom peak. (TIFF 189 KB)

Additional file 8: **Alternative promoter usage in** ***Tnfrsf12a*** **gene transcription after ONC.** Genome Explorer view comparing the distribution of mapped CAGE tags for the *Tnfrsf12a* gene in the ONC and sham groups. The X-axis indicates the number of mapped CAGE tags. Alternative promoters of *Tnfrsf12a* were activated at the bottom peak after ONC (red frame). (TIFF 212 KB)

Additional file 9: **Detailed list of functions and molecules associated with the “Cell Death and Survival” pathway after ONC.** (XLSX 51 KB)

Additional file 10: **List of DEGs involved in the Cell death and Cell survival pathways after ONC.** (XLSX 35 KB)

Additional file 11: **Predicted regulators of axonal injury after ONC.** (PDF 46 KB)

Additional file 12: **DNA sequence motifs likely to regulate gene expression changes after ONC.** Logos of the most significantly up- or down-regulated DNA sequence motifs for each TSS peak are shown. The relative size of the letters represents their frequency in the motif sequences. (TIFF 150 KB)
