# Supplementary figures and images for: Retinal transcriptome profiling at transcription start sites: a cap analysis of gene expression early after axonal injury
Source: BMC Genomics. 2014 Nov 18;15(1):982. doi: 10.1186/1471-2164-15-982 (PMC4246558; doi:10.1186/1471-2164-15-982)

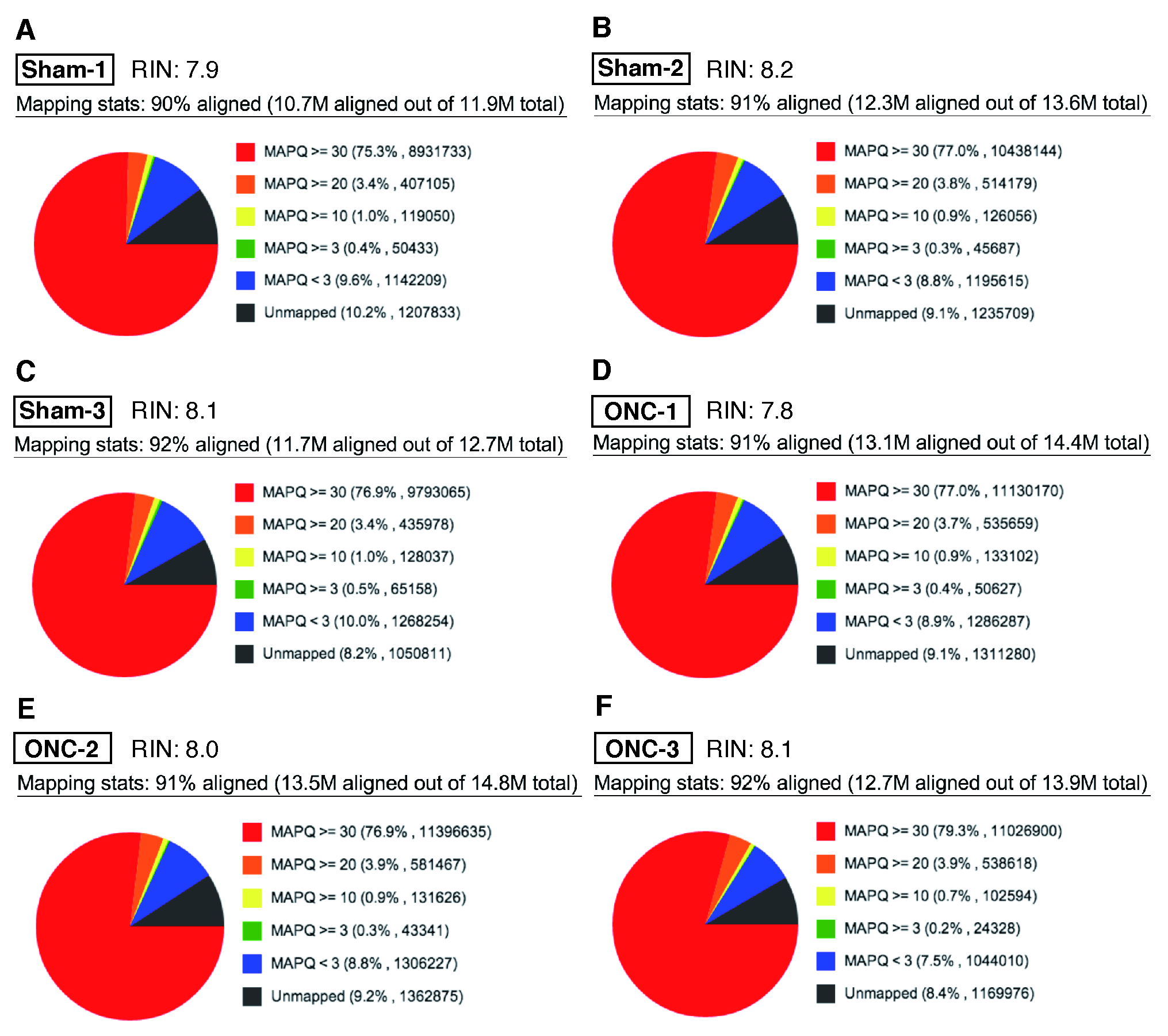

Supplement: Supplementary file 1 — Additional file 1: Summary of mapping statistics from SAMStat output. The pie charts show the number of sequence alignments in various mapping quality (MAPQ) intervals and the number of unmapped sequences. The percentage and number of alignments in each category is given in brackets. Red indicates reads with a high mapping accuracy (MAPQ > 30). Black indicates unmapped reads. The RNA integrity number (RIN) of each sample is shown after the sample name. (TIFF 1 MB) [file 12864_2014_6671_MOESM1_ESM.tiff]

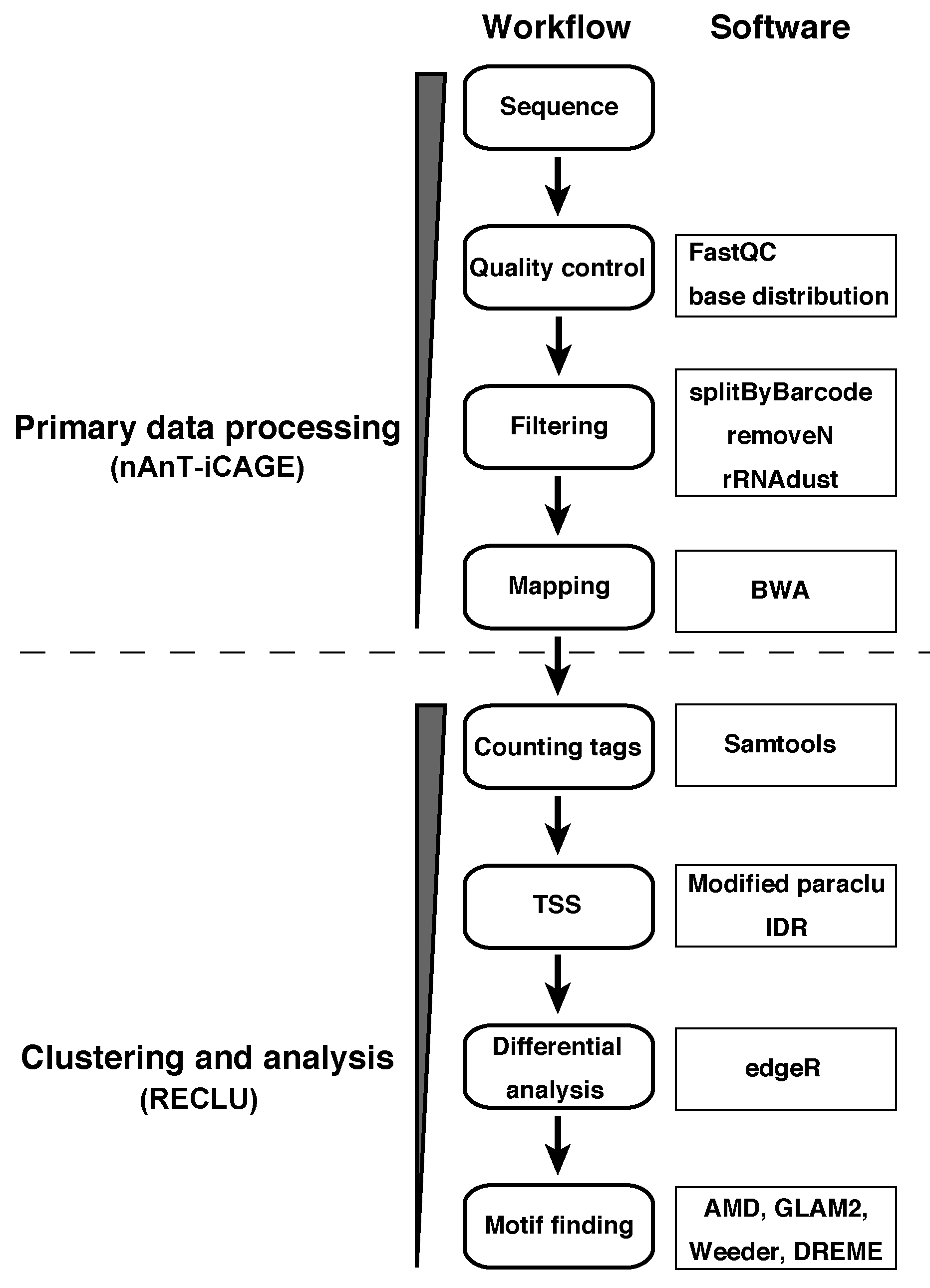

Supplement: Supplementary file 2 — Additional file 2: CAGE data analysis workflow. The workflow has two phases: primary data processing followed by clustering and analysis. During the first phase, the sequence data is evaluated for quality, filtered, mapped, and converted to an annotation file with a nAnT-iCAGE pipeline. During the second phase, the file is fed into the analytical pipeline, where the analysis of differential expression and motif discovery are carried out with the RECLU pipeline. (TIFF 163 KB) [file 12864_2014_6671_MOESM2_ESM.tiff]

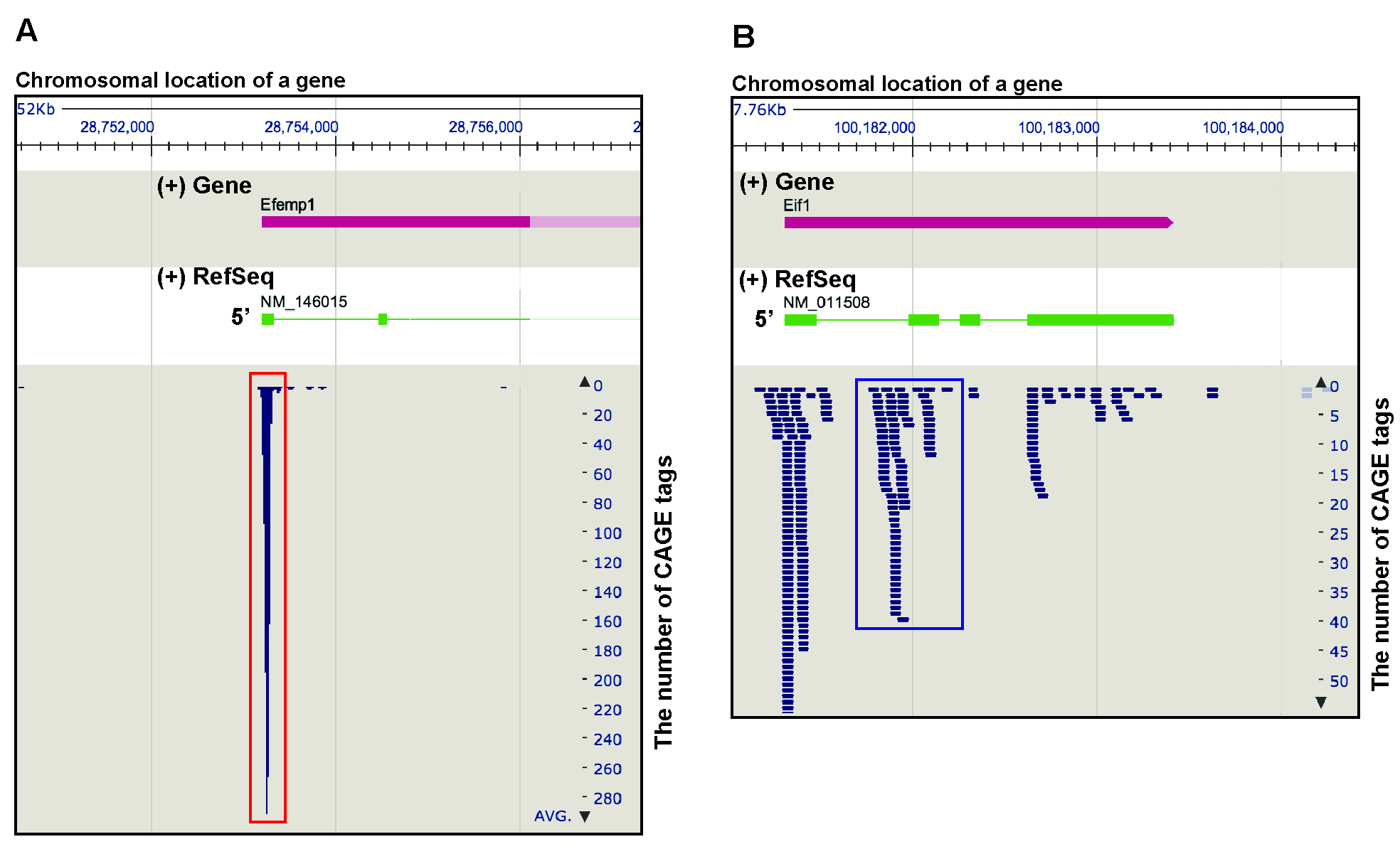

Supplement: Supplementary file 7 — Additional file 7: Example of top and bottom peaks of TSS clusters. Genome Explorer views of Efemp1 and Eif1 genes are shown here, representing TSS clusters with a top peak (A) and a bottom peak (B), defined with a modified Paraclu program. Based on the distribution of the mapped tags, TSS clusters show two types of structures, sharp with a high peak (termed “top”) and broad with a low peak (termed “bottom”) [42]. The red frame indicates a top peak. The blue frame indicates a bottom peak. (TIFF 189 KB) [file 12864_2014_6671_MOESM7_ESM.tiff]

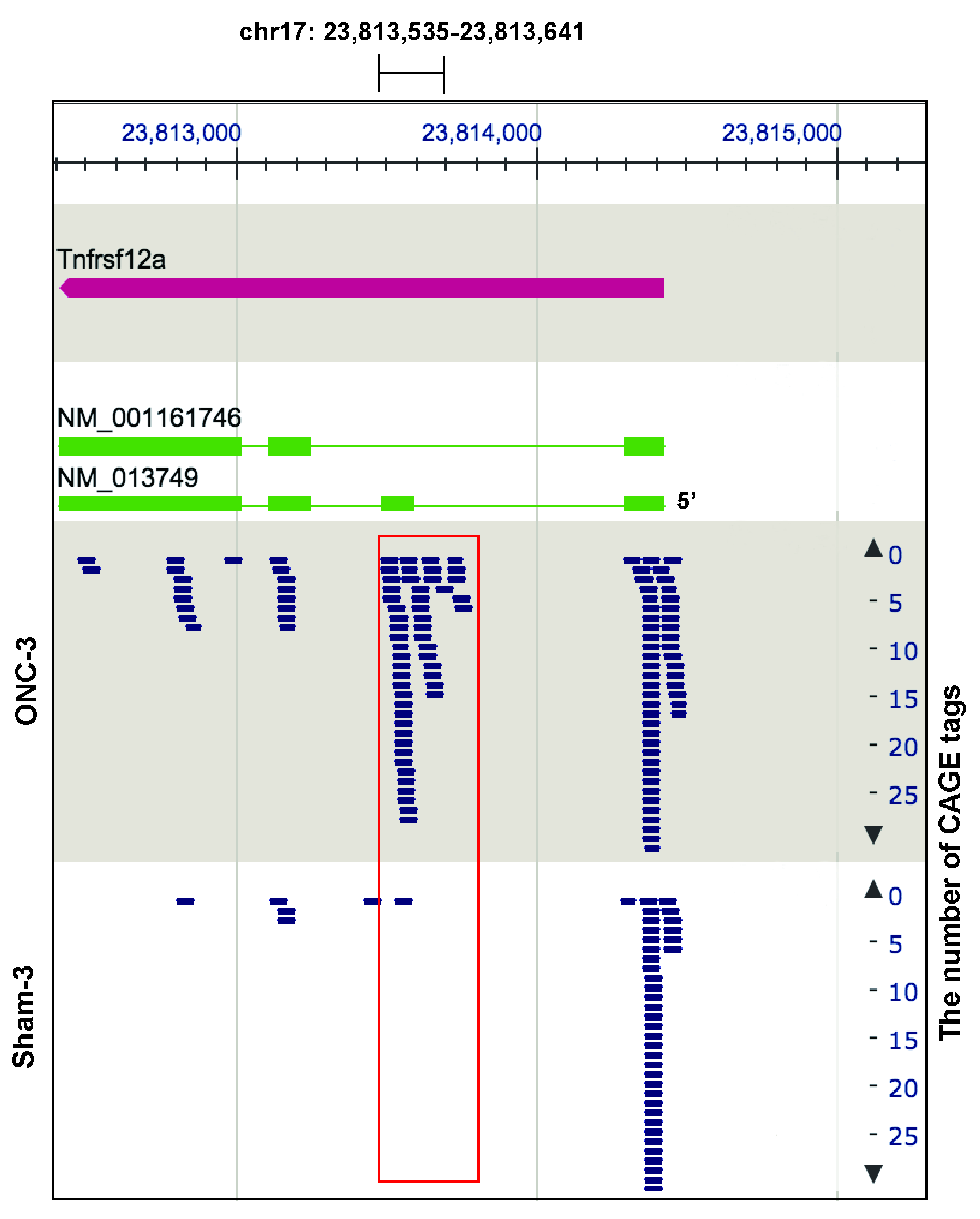

Supplement: Supplementary file 8 — Additional file 8: Alternative promoter usage in Tnfrsf12a gene transcription after ONC. Genome Explorer view comparing the distribution of mapped CAGE tags for the Tnfrsf12a gene in the ONC and sham groups. The X-axis indicates the number of mapped CAGE tags. Alternative promoters of Tnfrsf12a were activated at the bottom peak after ONC (red frame). (TIFF 212 KB) [file 12864_2014_6671_MOESM8_ESM.tiff]

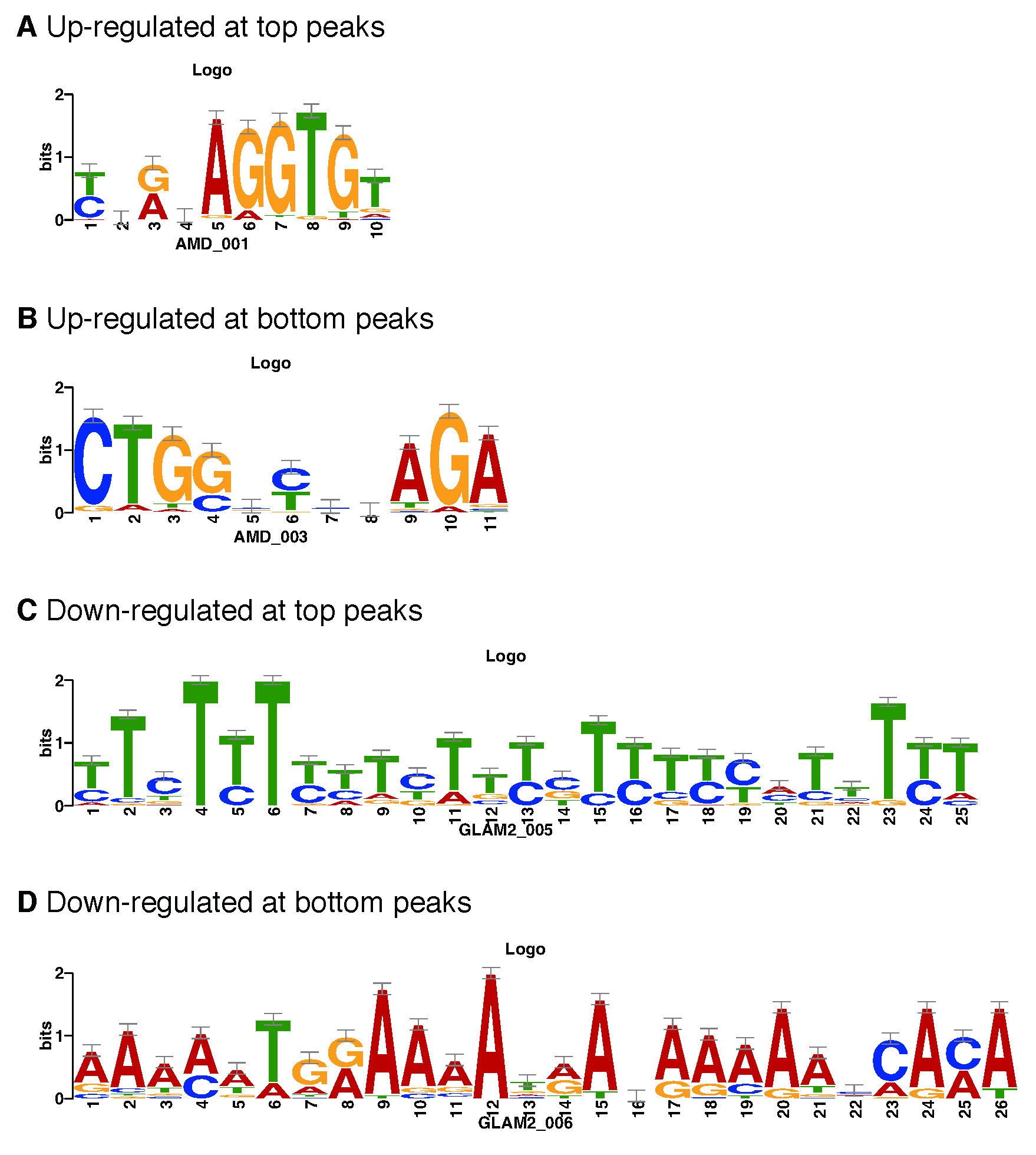

Supplement: Supplementary file 12 — Additional file 12: DNA sequence motifs likely to regulate gene expression changes after ONC. Logos of the most significantly up- or down-regulated DNA sequence motifs for each TSS peak are shown. The relative size of the letters represents their frequency in the motif sequences. (TIFF 150 KB) [file 12864_2014_6671_MOESM12_ESM.tiff]
