# Supplementary material for: Retinal transcriptome profiling at transcription start sites: a cap analysis of gene expression early after axonal injury
Source: BMC Genomics. 2014 Nov 18;15(1):982. doi: 10.1186/1471-2164-15-982 (PMC4246558; doi:10.1186/1471-2164-15-982)
Supplement: Supplementary file 3 — Additional file 3: List of Taqman probes used in this study. (PDF 44 KB) [file 12864_2014_6671_MOESM3_ESM.pdf]

Additional file 3: List of Taqman probes used in this study.

Gene symbol	Assay ID
Bcat1	Mm01259194_m1
Cox6a2	Mm01278190_g1
Crabp2	Mm00801691_m1
Fxyd7	Mm00469662_m1
Gng4	Mm01160620_m1
Tppp3	Mm00471425_m1
Gapdh	Mm99999915_g1
