# Supplementary material for: Retinal transcriptome profiling at transcription start sites: a cap analysis of gene expression early after axonal injury
Source: BMC Genomics. 2014 Nov 18;15(1):982. doi: 10.1186/1471-2164-15-982 (PMC4246558; doi:10.1186/1471-2164-15-982)
Supplement: Supplementary file 11 — Additional file 11: Predicted regulators of axonal injury after ONC. (PDF 46 KB) [file 12864_2014_6671_MOESM11_ESM.pdf]

Additional file 11. Predicted regulators of axonal injury after ONC.

Regulators	Consistency score	Diseases & Functions	Target molecules in dataset
IFNG, P38 MAPK, TP53	5.82	apoptosis of fibroblasts, cell death of fibroblast cell lines, necrosis of muscle	ATF3, BBC3, BCL2, CDKN1A, CEBPB, CLIC4, DDIT3, EGR1, HMOX1, JUN, KITLG, PARK7, SPRR1A
IFNG	-6.00	nephritis	BCL2, CDKN1A, CEBPB, FCGR2B

The consistency score is a measure of the causal consistency and the connection density of a regulator effects network.
